# Effects of visual short-term memory load and attentional demand on the contrast response function

**DOI:** 10.1167/jov.20.10.6

**Published:** 2020-10-02

**Authors:** Nikos Konstantinou, Nilli Lavie

**Affiliations:** 1 Department of Rehabilitation Sciences, Cyprus University of Technology, Limassol, Cyprus; 2 Institute of Cognitive Neuroscience, University College London, UK

**Keywords:** visual short-term memory, working memory, attention, perceptual load, contrast response function

## Abstract

Visual short-term memory (VSTM) load leads to impaired perception during maintenance. Here, we fitted the contrast response function to psychometric orientation discrimination data while also varying attention demand during maintenance to investigate: (1) whether VSTM load effects on perception are mediated by a modulation of the contrast threshold, consistent with contrast gain accounts, or by the function asymptote (1 lapse rate), consistent with response gain accounts; and (2) whether the VSTM load effects on the contrast response function depend on the availability of attentional resources. We manipulated VSTM load via the number of items in the memory set in a color and location VSTM task and assessed the contrast response function for an orientation discrimination task during maintenance. Attention demand was varied through spatial cuing of the orientation stimulus. Higher VSTM load increased the estimated contrast threshold of the contrast response function without affecting the estimated asymptote, but only when the discrimination task demanded attention. When attentional demand was reduced (in the cued conditions), the VSTM load effects on the contrast threshold were eliminated. The results suggest that VSTM load reduces perceptual sensitivity by increasing contrast thresholds, suggestive of a contrast gain modulation mechanism, as long as the perceptual discrimination task demands attention. These findings support recent claims that attentional resources are shared between perception and VSTM maintenance processes.

## Introduction

Visual short-term memory (VSTM), also termed visual working memory (e.g., Luck & Vogel, 1997), links perception with higher cognitive functions via maintenance of visual information for short periods of time (Fukuda, Awh, & Vogel, 2010; Johnson et al., 2013; Luck & Vogel, 2013). For example, when we play a sports game such as basketball, we rely on VSTM to maintain the position of each player and the referees on the court before we decide our next move with the ball. Yet, as it is so often evidenced in sports games, we experience failures of VSTM when the information we need to maintain exceeds our capacity limits (for example, when trying to decide whether a particular formation is critical for a shoot). Much research has shown that VSTM has limited capacity, whether these limits are modeled as a limited number of “slots” or as processing limitations (Conway, Cowan, & Bunting, 2001; Cowan, 2010; Franconeri, Alvarez, & Cavanagh, 2013; Luck & Vogel, 1997; Ma, Husain, & Bays, 2014; Todd & Marois, 2004; Woodman, Vogel, & Luck, 2001).

A number of studies have demonstrated that loading VSTM reduces both distraction and detection sensitivity for a visual stimulus presented during the memory delay (Konstantinou, Beal, King, & Lavie, 2014; Konstantinou & Lavie, 2013). VSTM load was also found to reduce the retinotopic response to a contrast increment presented during the maintenance delay in early visual cortex areas V1 to V3 (Konstantinou, Bahrami, Rees, & Lavie, 2012). These effects are in line with the sensory recruitment hypothesis (e.g., Serences, Ester, Vogel, & Awh, 2009; for more recent formulations, see Gayet, Paffen, & Van der Stigchel, 2018; Scimeca, Kiyonaga, & D'Esposito, 2018), which suggests that the brain network responsible for maintenance of visual information in memory involves the same sensory brain areas as those involved in perceptual encoding. The reduction of the V1 to V3 response to stimuli and accompanied findings of reduced detection sensitivity during the maintenance interval in conditions of higher VSTM load can be taken to reflect that loading VSTM depletes the sensory resources required for perceptual representations of incoming stimuli during maintenance.

It remains unclear, however, whether the effects of VSTM load on perception are directly due to VSTM engaging the sensory resources required for visual perception (due to sensory recruitment in memory maintenance) (e.g., Serences et al., 2009) or whether they may also depend on VSTM engaging attentional resources that are critical for perception and the mediated sensory processing. It may also be possible to propose that the effects are merely due to a change in the top–down bias such that detection responses to stimuli that are irrelevant to those maintained in VSTM are deprioritized when the VSTM task is more demanding (under conditions of high VSTM load). We addressed these questions by investigating the effects of VSTM load on the contrast response function and testing the role of attentional demand during maintenance in these effects. Below, we briefly review the relevant previous research.

### Contrast gain versus response gain effects

The question of whether VSTM load affects the contrast response function via contrast gain, response gain, or a combination of both is important because the different effects are thought to reflect different underlying neural mechanisms. Neurons in the visual cortex exhibit a systematic nonlinear increase in firing rate with increasing stimulus contrast, evidenced in the contrast response function (Albrecht & Hamilton, 1982; Gardner, Sun, Waggoner, Ueno, Tanaka, & Cheng, 2005; Sclar, Maunsell, & Lennie, 1990). A similar pattern is observed in psychophysical performance (Braun, 1998; Carrasco, 2006; Sperling & Melchner, 1978), which can thus be used to draw conclusions about the underlying neural responses to contrast stimuli (Pestilli, Ling, & Carrasco, 2009). Using logic similar to that of Ling and Carrasco (2006; cf. Pestilli et al., 2009), we draw inferences regarding contrast gain and response gain from fits of (neural) contrast response functions to response data.

Because, to the best of our knowledge, the present work is the first to establish the effects of VSTM load on the contrast response function, we have used a traditional psychophysics model that relates behavioral discrimination accuracy to contrast, while not including additional components to model the putative single neuron responses (cf. May & Solomon, 2015) for the sake of simplicity, in an attempt to test the relationship of VSTM and contrast perception. Nevertheless, in order to understand the fundamental mechanisms of contrast gain versus response gain, it is important to outline the underlying neural mechanisms; therefore, we briefly discuss these below.

#### Contrast gain effects

**Figure 1.**
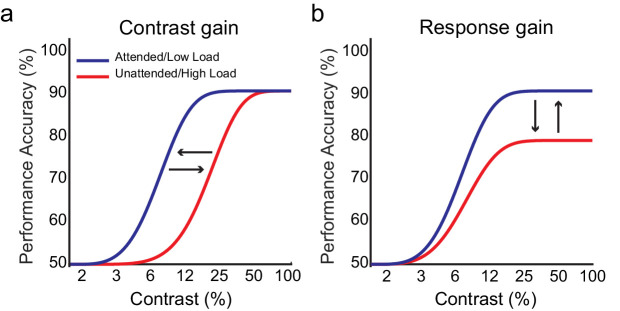
Possible effects of attention and VSTM load on the contrast response function. (a) The contrast gain account predicts that attention will increase but load will decrease contrast sensitivity, thus resulting in a decrease of the effective contrast of a visual stimulus. The contrast gain effect of load is characterized by a rightward shift of the function in the high load condition (red curve) compared to the low load condition (blue curve) without any change in the asymptote of the function. (b) The response gain model predicts that attention will increase but load will decrease the response to stimulus contrast, which is characterized by a change in the asymptote of the contrast response function.

A contrast gain effect is reflected in the contrast response or psychometric functions as a gain multiplication on the effective strength of sensory input (e.g., Martínez-Trujillo & Treue, 2002; Reynolds, Pasternak, & Desimone, 2000; Schwedhelm, Krishna, & Treue, 2016), which therefore results in a horizontal shift of the function (Figure 1a). When considering the underlying neural mechanism for a contrast gain modulation by higher level cognitive factors such as VSTM load or attention (as opposed to actual stimulus input factors), a contrast gain modulation is proposed to reflect an interactive modulation of the stimulus-evoked response in sensory visual cortex neurons, reflecting a modulation of their sensitivity during their processing of the stimulus contrast and thus making it appear as a change in the effective strength of the sensory input in the contrast response function (e.g., Ling & Carrasco, 2006). Thus, the sensory recruitment hypothesis of VSTM leads to the prediction of a contrast gain effect of VSTM load, suggesting that the higher sensory recruitment in conditions of high VSTM load depletes the sensory resources critical for processing a stimulus contrast during maintenance.

#### Response gain effects

Response gain effects reflect a multiplication of the neural responses to contrast (i.e., the output rather than the effective input) by a fixed gain factor that is independent of the neural processing of contrast (McAdams & Maunsell, 1999; Treue & Martínez Trujillo, 1999), simply adding a fixed additive increase of the response to the same stimulus (resulting in a vertical shift of the asymptote) (Figure 1b). An effect of VSTM load on the response gain can therefore be explained as the result of a top–down bias, reflecting reduced prioritization of responses to secondary task (detection) stimuli as the primary VSTM task becomes more demanding in conditions of higher load (e.g., a form of goal neglect due to the greater demand in the primary task) (Duncan, Emslie, Williams, Johnson, & Freer, 1996).

### VSTM and attention

Although much previous work has demonstrated the link between attention and VSTM encoding (e.g., Adam, Mance, Fukuda, & Vogel, 2015; Giesbrecht, Weissman, Woldorff, & Mangun, 2006; Murray, Nobre, & Stokes, 2011; Myers, Stokes, Walther, & Nobre, 2014; Robison & Unsworth, 2019), more recent work has demonstrated that performance of VSTM and visual attention tasks is characterized by synchronous fluctuations over time (Balestrieri, Ronconi, & Melcher, 2019; deBettencourt, Keene, Awh, & Vogel, 2019). These findings are taken to support the idea that attentional resources are shared between perceptual representations of visual information and visual information maintained in VSTM (Adam & deBettencourt, 2019). It is therefore possible that the effects of VSTM load on detection and the associated neural responses are mediated by greater demands on attentional resources during high load maintenance. If this is the case, then any effects of VSTM load on the contrast response function should manifest via contrast gain, indicating reduced sensitivity due to depletion of attentional resources (e.g., Lavie, Beck, & Konstantinou, 2014), but, critically, these should also depend on the level of the attentional demands involved in the detection task during VSTM maintenance.

### The present research

In the present study, we set out to examine whether the effects of VSTM load on the contrast response function are accounted for by contrast gain, response gain, or a combination of both (Experiment 1). Next, we investigated whether the effects of VSTM load on the contrast response function depend on the availability of attentional resources by varying attentional demands during the maintenance delay of a VSTM task (Experiment 2). To this purpose we calculated the effects of low and high VSTM load on the contrast response function relating orientation discrimination accuracy to the contrast of a stimulus presented during the memory delay.

We hypothesized that if the effects of impaired perception with VSTM load are due to VSTM load impacting directly on sensory perception, as implied by our previous findings of VSTM load effects on detection sensitivity and on stimulus-evoked activity on retinotopic visual cortex (V1–V3) during maintenance (Konstantinou et al., 2012; Konstantinou & Lavie, 2013), then such effects should affect neural sensitivity to contrast, resulting in a shift of the contrast response function to the right (Figure 1a) in line with contrast gain effects. If the effects reflect a deprioritization of secondary (detection) task responses, then this should be manifested via response gain (Figure 1b). Deprioritization of task responses can reduce neural response overall by a fixed multiplicative factor that applies across the contrast response function in a manner that would not interact with the stimulus contrast (unlike the case for contrast gain effects). Importantly, if VSTM load exerts its effects via deprioritization of the secondary detection task response during maintenance and the observers fully prioritize the primary (VSTM) task rather than following instructions to flexibly allocate some resources to the secondary task—for example, 80% and 20% allocation between the primary and secondary tasks (e.g., Bonnel & Hafter, 1998)—then VSTM load effects should not interact with the secondary task demand on attention. In this case, the deprioritization bias should be driven by the demand of the primary (VSTM) task superimposed on secondary task performance (rather than interacting with the secondary task demands on attention).

To test these predictions, we combined an orientation discrimination task (in which the contrast of the orientation stimulus was varied) within the maintenance interval of a VSTM task of differing levels of load, and we instructed observers to fully prioritize the memory task performance accuracy. We then quantitatively estimated the impact of VSTM load on the contrast response function. Load effects on the estimated contrast threshold (the stimulus contrast at half the maximum performance) between the two load conditions would indicate a horizontal shift of the contrast response function and support interpretation of the effects as a contrast gain. Load effects on the asymptote (response saturation level) of the contrast response function would indicate response gain effects (Ling & Carrasco, 2006).

## Experiment 1

In Experiment 1, we asked whether VSTM load affects the perception of an unattended stimulus contrast via an effect on the contrast gain, the response gain, or their combination. Participants performed a VSTM task that required maintenance of either the color of a single square (low load) or the conjunction of color and location of a set of four squares (high load) (e.g., Konstantinou et al., 2012; Luck & Vogel, 1997). During the VSTM task, participants also engaged in an orientation discrimination task that required discrimination of the tilt angle (clockwise or counterclockwise) of a Gabor patch presented in the periphery. The orientation discrimination task was presented during the delay period of the VSTM task. The increased memory set size under the high load condition increased demands on visual maintenance because a greater amount of visuospatial information had to be maintained during the memory delay.

### Method

#### Participants

Twelve right-handed individuals (four males; mean age, 26.8 years; age range, 18–34 years) participated in Experiment 1. Three participants were replaced because of poor memory performance (all three memory estimates using Cowan's *K* in high load < 1.2). Another participant was replaced because a software failure resulted in the loss of the participant's responses during the experiment. All participants were treated in accordance with the tenets of the Declaration of Helsinki.

#### Stimuli and apparatus

**Figure 2.**
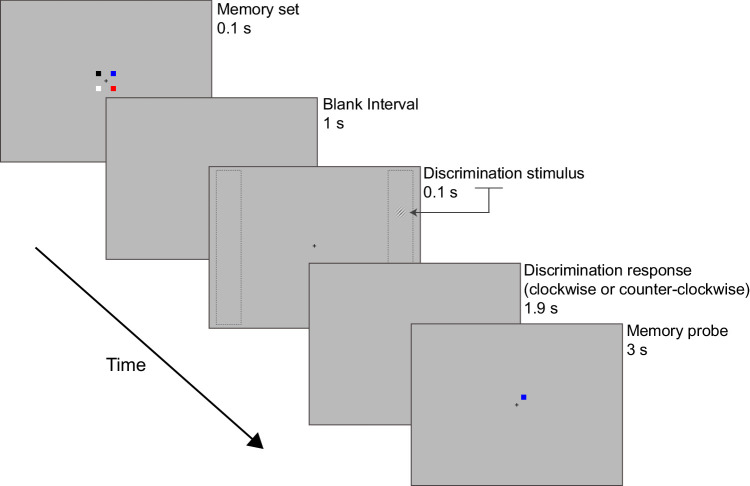
Displays of an example high VSTM load trial in Experiment 1. Four squares in high load (or one square in low load) were presented at the center of the screen. Following a 1-second interval, a tilted target Gabor patch appeared within a left or right columnar bar (shown as gray dashed lines here but not presented during the experiment). Participants maintained the memory set while reporting (during the delay period) the orientation (clockwise or counterclockwise) of the peripheral Gabor patch. The correct response here is counterclockwise. The memory probe appeared next for 3 seconds or until response. Participants indicated with a button press whether the location and color of the memory probe matched that of the memory set. The correct response here is “same.”

The experiment was presented on a personal computer attached to a 20-inch cathode-ray tube monitor (resolution, 800 × 600 pixels; refresh rate, 60 Hz; mean background luminance, ∼70 cd/m^2^) and a standard QWERTY keyboard. The experiment was programmed and presented using the Cogent toolbox (www.vislab.ucl.ac.uk/cogent.php) for MATLAB (MathWorks, Inc., Natick, MA). Figure 2 illustrates the stimuli and trial sequence. The memory set contained either one (low load) or four (high load) colored squares (0.38° × 0.38°) randomly placed on a 3 × 3 grid (1.38° × 1.38°) centered at fixation. Each square was of a different color, chosen randomly from black (<0.01 cd/m²), blue (*x* = 0.15, *y* = 0.07; 29.05 cd/m²), cyan (*x* = 0.20, *y* = 0.27; 69 cd/m²), green (*x* = 0.27, *y* = 0.59; 65.84 cd/m²), magenta (*x* = 0.28, *y* = 0.14; 48.20 cd/m²), pink (*x* = 0.32, *y* = 0.30; 69.14 cd/m²), red (*x* = 0.62, *y* = 0.33; 39.56 cd/m²), white (77 cd/m²), and yellow (*x* = 0.40, *y* = 0.49; 73.61 cd/m²). The memory probe was a single square presented on the location of the memory set item in low load and on one of the occupied memory set positions in high load.

For the orientation discrimination task, a Gabor patch (sinusoidal grating of 3 cpd enveloped in a Gaussian window, tilted left or right) was presented within a left or right columnar bar (vertical length, 12.4°; horizontal eccentricity from midline, 6.2°) in a counterbalanced fashion, with the exact location within the columnar bar randomly assigned. Prior to the main experiment, the tilt angle of the Gabor patch was individually assessed for each participant. A staircase procedure was implemented using an accelerated stochastic approximation method to obtain the tilt angle estimate that resulted in approximately 75% accuracy rate (Kesten, 1958; Lavie et al., 2014). This ensured that, when assessing the contrast response function, the tilt angle would be difficult enough to avoid ceiling performance even at maximum contrast, thus allowing us to achieve a lower than 100% asymptote, which is essential for measuring any potential response gain effects. In the main experiment, the full contrast response function was estimated using the method of constant stimuli. The Gabor contrast was randomly chosen in each trial from a set of eight contrasts (0.1%, 7.3%, 14.4%, 21.6%, 35.9%, 43.1%, 66.5%, and 90%).

#### Procedure

The experimental procedure is illustrated in Figure 2. All trials were initiated by the participant via button press. A fixation mark appeared first for 1 second followed by the VSTM set display for 100 ms that contained one colored square for low load and four colored squares for high load. Following a 1-second blank interval, the orientation discrimination task with the tilted Gabor in the periphery was presented. Participants were then given 1.9 seconds to respond with their left hand as to whether the Gabor was tilted clockwise (index finger) or counterclockwise (middle finger). Next, the memory probe appeared for 3 seconds (or until response) and was comprised of one colored square in the location of one of the memory set items. Participants indicated with a right-hand button press whether the location and color of the memory probe matched those of the memory set (index finger indicated “same”; middle finger indicated “different”). The memory probe matched the memory set in half of the trials, whereas it changed color during the other, unmatched half.

Feedback was given only on incorrect memory responses with the words “WRONG memory response” appearing above fixation. Responses to the two tasks were not timed. Participants completed six 64-trial runs (following one practice run), resulting in a total of 384 trials (192 trials per load condition). Each run contained eight blocks of eight trials each, with the block order ABBABAAB in each run, counterbalanced across participants. Prior to the main task, participants were instructed that their main task priority was to respond as accurately as possible to the VSTM task. They were shown example displays of the experiment, and the experimenter stressed this task priority as these were shown. After ensuring that participants clearly understood task priorities, they proceeded to the practice run and full experiment.

#### Contrast response function

To assess whether the effects of VSTM load on visual perception are consistent with contrast gain or response gain, the data from each participant (i.e., orientation discrimination accuracy data from trials with a correct VSTM response) were fitted to the Naka–Rushton model, which has been previously shown to describe well the relationship between the contrast of a visual stimulus and neural response either on the basis of single neuron research (Albrecht & De Valois, 1981; Albrecht & Hamilton, 1982; Naka & Rushton, 1966) or on the basis of behavioral psychophysics (Ling & Carrasco, 2006; Pestilli et al., 2009):

$$\psi \left(x;\alpha ,\beta ,\gamma ,\lambda \right)=\gamma +\left(1-\gamma -\lambda \right)F\left(x;\alpha ,\beta \right)$$

where *x* is the stimulus contrast; *α*, *β*, *γ*, and *λ* are the fitted model parameters that determine the shape of the contrast response function; and *F* is the Naka–Rushton function:

$$\begin{array}{ccc} & & F\left(x;\alpha ,\beta \right)=x^{\beta }/\left(x^{\beta }+\alpha^{\beta }\right),\\ & & \mathrm{with}\;x\;\in \left(-\infty ,\;+\infty \right),\;\alpha \;\in \left(-\infty ,+\infty \right) \end{array}$$

The contrast threshold (*α*) and the asymptote (*λ*) of the contrast response function were left to vary freely and were estimated separately for the low and high load conditions. Because we aimed to focus on effects on the contrast threshold (*α*) reflecting contrast gain and the asymptote (1 – *λ*) reflecting response gain, we forced the two other parameters of guess rate (*γ*), and slope (*β*) to be the same for both low load and high load conditions. The *γ* parameter was set at 0.5 to reflect chance performance in the orientation discrimination task. The *β* parameter was set at 2, estimated by collapsing all individual data into a single pool and fitting the pooled data to the Naka–Rushton model.

Fits were performed using maximum likelihood estimation, and the errors of the parameters were estimated by parametric bootstrap analysis. Goodness of fit was assessed with deviance scores, which were calculated as the log-likelihood ratio between a fully saturated model and the data model. This analysis confirmed good fits in all participants, as indicated by cumulative probability estimates of the obtained deviance scores (all *p* > 0.05).

### Results

#### Visual short-term memory

Task accuracy decreased significantly from the low (*M* = 94%, *SD* = 3%) to the high (*M* = 69%, *SD* = 10%) VSTM load condition, *t*(11) = 9.68, *p* < 0.001, *d* = 1.70. The amount of information estimated to be maintained in VSTM using Cowan's *K* (Cowan, Elliott, Saults, Morey, Mattox, Hismjatullina, & Conway, 2005)—*K* = *N*(hit rate – false alarm rate), where *K* is the memory estimate and *N* is the number of items presented in the memory set—was significantly increased from the low (*K* = 0.89, *SD* = 0.05) to the high (*K* = 1.53, *SD* = 0.78) VSTM load condition, *t*(11) = 2.85, *p* = 0.02, *d* = 0.99. These results demonstrate that the VSTM load manipulation in Experiment 1 was effective and participants held more information in VSTM during the high versus the low load condition.

#### Contrast response function

**Figure 3.**
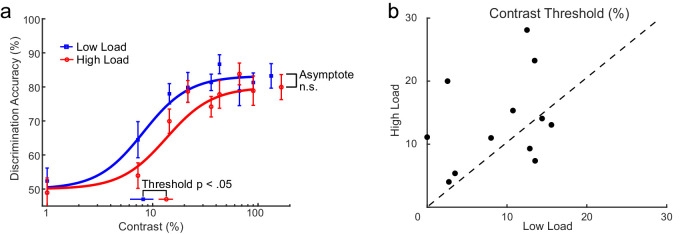
Effects of VSTM load on orientation discrimination accuracy performance in Experiment 1. (a) Contrast response functions for low (blue curve) and high (red curve) VSTM load. The estimated contrast threshold parameter for each contrast response function is also shown (contrast threshold yielding half-maximum performance). Each function was generated by averaging parameter values across participants (separately for low and high load). Each data point represents the mean across participants. Error bars are ±1 *SEM*. (b) The estimated contrast threshold of individual participants plotted for high versus low VSTM load.

Figure 3a depicts the group average contrast response functions for the low and high VSTM load and their Naka–Rushton fits as implemented in the Palamedes toolbox (Prins & Kingdon, 2009). As predicted by the contrast gain account, the function of the high VSTM load condition was shifted to the right compared with the low load condition. In addition, no differences in the asymptotic performance between the two conditions were observed.

The estimated individual contrast thresholds from the Naka–Rushton fits are depicted in Figure 3b for each participant. As predicted by contrast gain, high (vs. low) VSTM load led to a significant increase in the contrast threshold (low load: *M* = 8%, *SD* = 6%; high load: *M* = 15%, *SD* = 9%), *t*(11) = –2.28, *p* = 0.04, *d* = –0.66. No evidence for a reliable change in the asymptote of the contrast response function was found (low load: *M* = 84%, *SD* = 9%; high load: *M* = 81%, *SD* = 9%), *t*(11) = 1.20, *p* = 0.26, *d* = 0.35. These results demonstrate a rightward shift in the contrast response function with higher VSTM load. Importantly, the asymptote of the contrast response function is not affected by VSTM load. This pattern of results is characteristic of the effects of contrast gain.

To further test whether contrast gain is the best model fit for the individual data as suggested by the average results, we computed Akaike's information criterion (AIC; Akaike, 1974) as a means for model selection that balances relative goodness of fit with model simplicity. The models considered were the following: Model 1 was a contrast gain model (as suggested by the average results), in which both the thresholds and asymptotes were free to vary but the asymptotes were constrained to take on the same value in low and VSTM high load conditions. Model 2 was a response gain model, in which thresholds were constrained to take on the same value in the low and high VSTM load conditions and asymptotes were allowed to differ between low and high VSTM loads. Model 3 was a combined contrast gain and response gain model, in which both thresholds and asymptotes differ between low and high VSTM load, and Model 4 was a fully constrained model, in which thresholds and asymptotes were free to vary but constrained to take on the same value in low and high VSTM load conditions. Based on this analysis, the AIC criterion indicated that Model 1 provided a better fit of the data compared to the rest of the models for nine out of 12 participants (see Table 1) in support of a contrast gain account for the effects of VSTM load.

**Table 1.** ΔAIC values for the four models tested for Experiment 1. *Notes*: For each participant, the difference (Δ) in AIC values is shown compared to the best-fitting model for that participant. ΔAIC values for the best-fitting model are zero (presented in bold).

	ΔAIC			
Participant	Model 1	Model 2	Model 3	Model 4
1	**0.00**	1.09	1.90	3.16
2	**0.00**	0.13	0.15	6.18
3	**0.00**	1.29	1.92	3.36
4	**0.00**	1.14	2.00	3.75
5	**0.00**	0.61	1.97	2.63
6	0.89	**0.00**	1.92	2.89
7	**0.00**	0.29	2.00	2.36
8	0.06	**0.00**	1.87	2.08
9	**0.00**	1.98	0.88	4.19
10	**0.00**	0.07	1.95	2.28
11	**0.00**	2.00	2.00	4.00
12	0.71	**0.00**	1.01	6.81

## Experiment 2

Experiment 1 findings that VSTM load reduced contrast gain support the prediction that VSTM load affects sensory contrast perception. In Experiment 2, we tested whether these effects depend on attention. To this purpose, we modified the design of the task used in Experiment 1 by varying the demand placed on attention by the orientation discrimination task by manipulating the spatial uncertainty of the orientation stimulus.

**Figure 4.**
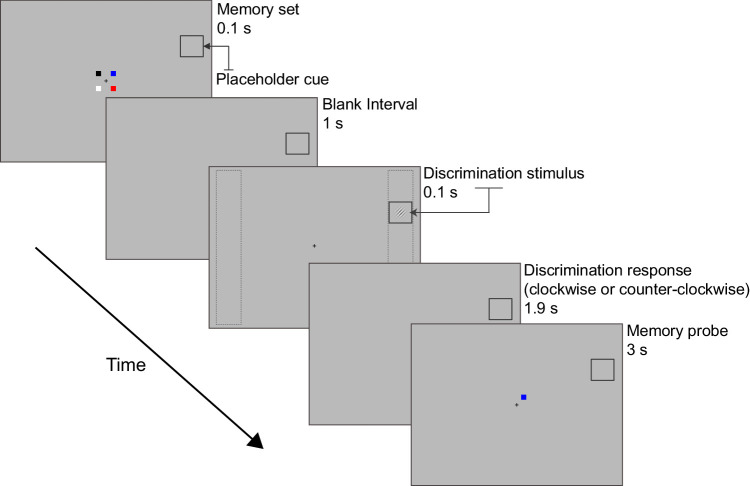
An example of a high VSTM load trial in the certain condition in Experiment 2. The procedure was identical to that of Experiment 1 except that a placeholder (see top right corner of the stimulus displays here) that indicated the spatial location of the Gabor patch was present throughout the duration of each trial. As in Experiment 1, the Gabor patch was presented inside a left or right columnar bar, shown here as gray dashed lines in the discrimination stimulus display but not presented during the experiment.

In a 2 × 2 design, VSTM load was manipulated by varying the number of items in the memory set (as before, low load had one item and high load had four items) and attention was manipulated through the presence (certain condition) or absence (uncertain condition) of a placeholder (spatial cue). In the certain condition, a placeholder cue was present throughout each trial within which the target stimulus appeared during the memory delay. The presence of the placeholder indicated the spatial location of the stimulus in all trials and thus reduced competition for attention to a spatial area roughly the same size as that of the target. However, in the absence of the placeholder, attention spread throughout a larger spatial field around fixation (Figure 4), as the spatial location of the stimulus was uncertain (as was the case in Experiment 1).

The specific modification of the task employed in Experiment 2 was directly related to the normalization model of attention (Reynolds & Heeger, 2009), according to which the effects of attention on contrast response function depend on the size of the stimulus and the size of the attention field. When the stimulus is small and the attention field is large, the model predicts contrast gain changes. This was the case in Experiment 1 and in the uncertain condition of Experiment 2, where the orientation discrimination stimulus appeared with high spatial uncertainty during the maintenance interval, thus the visual perception task demanded attentional resources to resolve the location of the stimulus. In contrast, when the stimulus is large and the attention field small, as is the case in the certain condition in Experiment 2, the normalization model predicts that attention will affect contrast sensitivity via response gain (Herrmann, Montaser-Kouhsari, Carrasco, & Heeger, 2010). We therefore hypothesized that, if the effects of VSTM load interact with the availability of attentional resources, then the contrast gain effects of VSTM load would be observed only in the uncertain large attention field condition and not in the certain condition when the attention field is small relative to the stimulus size (which remained the same as in the uncertain condition).

If, however, the contrast gain effects with higher VSTM load of Experiment 1 are not due to increased demands on attentional resources, as we propose, but rather are solely due to increased demands on sensory resources (as per the sensory recruitment hypothesis) (Harrison & Tong, 2009; Rademaker, Chunharas, & Serences, 2019; Serences et al., 2009), then the effects of load should not interact with the spatial uncertainty of the target in the orientation discrimination task and should be observed in both the certain and uncertain conditions.

### Method

#### Participants

A separate group of 10 volunteers (four males; mean age, 23 years; age range, 18–30 years) took part in Experiment 2. One participant who failed to produce a reliable contrast response function (performance did not increase with contrast) for the entire experiment was replaced with a new participant.

#### Procedure

The stimuli and procedure were identical to those used in Experiment 1 except that in the certain condition the Gabor was presented within a 1.4° × 1.4° placeholder that was present throughout the entire duration of each trial (Figure 4). Participants completed 12 64-trial runs. The VSTM load condition and the uncertainty condition were blocked in eight-trial blocks presented in a counterbalanced fashion within each run.

#### Eye monitoring

We monitored fixation and eye blinks using infrared light transducers in the Skalar IRIS 6500 system (sampling rate, 1000 Hz; Skalar, Breda, The Netherlands) and recorded with DASYlab software (Measurement Computing, Norton, MA). Eye traces were recorded for a window of –100 to +200 ms around the orientation discrimination stimulus onset time on every trial. Online monitoring and offline trial-by-trial inspection of the data showed that participants managed to maintain fixation on >96% of all trials. Trials interrupted by eye blinks or eye movement during the measurement window were removed from analysis.

### Results

#### Visual short-term memory 

As expected, the VSTM task was of higher difficulty in the high compared to the low load condition, as confirmed by a two-way repeated-measures analysis of variance (ANOVA) on the memory task accuracies, with the factors VSTM load (low, high) and placeholder uncertainty (certain, uncertain). This analysis revealed a significant main effect of VSTM load (low load: *M* = 96%, *SD* = 1%; high load: *M* = 87%, *SD* = 7%), *F*(1, 9) = 29.32, *p* < 0.001, η^2^ = 0.77. The same analysis on the memory estimates (Cowan's *K*) also revealed a main effect of VSTM load (low load: *K* = 0.95, *SD* = 0.03; high load: *K* = 3.02, *SD* = 0.62), *F*(1, 9) = 118.05, *p* < 0.001, η^2^ = 0.93, indicating that participants indeed held more information in memory during the high (vs. low) VSTM load condition. These findings indicate effective manipulation of VSTM load. Importantly, the above analysis found no main effect of placeholder uncertainty (*F* < 1, for accuracy rates and for Cowan's *K* memory estimates) or interaction effects, *F*(1, 9) = 1.17, *p* = 0.31, η^2^ = 0.115 for accuracy rates, *F* < 1 for memory estimates. This result thus ensures that the spatial uncertainty manipulation did not affect VSTM task performance.

#### Contrast response function

**Figure 5.**
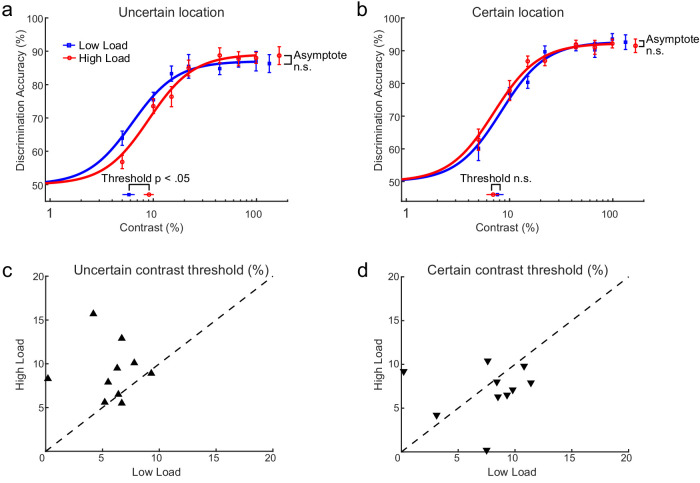
Effects of VSTM load on orientation discrimination accuracy (percent correct) in the certain and the uncertain target location conditions in Experiment 2. Shown are group-averaged contrast response functions of the low (blue curves) and the high (red curves) VSTM load for the uncertain (a) and certain (b) conditions. The estimated parameters from each contrast response function are also shown (contrast threshold yielding half-maximum performance; asymptotic performance). Each data point represents the mean across participants. Error bars are ±1 *SEM*. The bottom panels show the estimated contrast threshold of individual participants in the uncertain (c) and certain (d) conditions plotted for high versus low VSTM load. n.s., statistically non-significant.

Figure 5 shows the low and high VSTM load group average contrast response functions for the certain and uncertain conditions. Goodness-of-fit analysis indicated good fits for all participants (all *p* > 0.05). As predicted by the contrast gain account, when the spatial location of the discrimination stimulus was uncertain (i.e., there was no spatial cue), VSTM load led to an increase in the contrast threshold of the contrast response function, consistent with the findings of Experiment 1 (Figures 5a, 5c), with reliable differences in contrast threshold but no evidence for a change in the asymptote. Conversely, when the location of the discrimination stimulus was cued by the placeholder, no evidence for reliable differences in any of the estimated parameters was found (Figures 5b, 5d). Hence, in contrast to the consistent increase of contrast threshold with higher load observed when the stimulus location was uncertain, these findings demonstrate that the contrast gain effects of VSTM load were eliminated when spatial uncertainty was reduced, as is the case when attention demands on the orientation discrimination task are reduced by cuing the spatial location of the orientation stimulus.

Analyses on the parameter estimates from the individual data confirmed the findings from the group average data. A two-way repeated-measures ANOVA on contrast threshold estimates with the factors VSTM load (low, high) and placeholder uncertainty (certain, uncertain) revealed a significant interaction between VSTM load and placeholder uncertainty, *F*(1, 9) = 5.01, *p* = 0.05, η^2^ = 0.36. No main effects of either load or uncertainty were found; for load, *F*(1, 9) = 1.57, *p* = 0.24, η^2^ = 0.15; for uncertainty, *F* < 1. As can be seen in Figure 5, this interaction reflects the fact that contrast threshold estimates were increased under high VSTM load in the uncertain condition (low load: *M* = 6%, *SD* = 2%; high load: *M* = 9%, *SD* = 9%), *t*(9) = –2.50, *p* = 0.03, –*d* = 0.79, but not in the certain condition (low load: *M* = 8%, *SD* = 4%; high load: *M* = 7.5%, *SD* = 3%), *t*(9) = 0.50, p = 0.63, *d* = 0.16 (Figures 5c, 5d).

**Figure 6.**
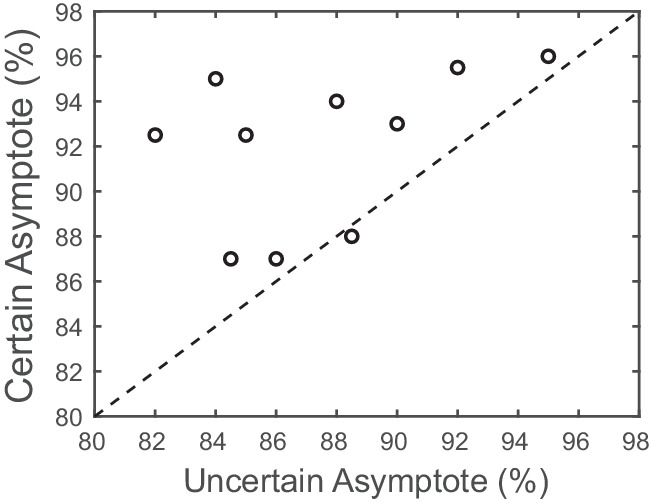
The effect of spatial cue on the orientation discrimination task asymptotic accuracy in Experiment 2. The plot depicts the estimated asymptote of individual participants, averaged across load, plotted for the certain versus uncertain condition.

In addition, a similar ANOVA on the estimated asymptote level of the individual contrast response functions revealed a significant main effect of placeholder certainty, *F*(1, 9) = 11.84, *p* < 0.01, η^2^ = 0.57. As shown in Figure 6, asymptotic performance was higher under the certain condition (*M* = 92%, *SD* = 3%) compared with the uncertain condition (*M* = 87%, *SD* = 5%), as predicted by the response gain account. There was no main effect of VSTM load (*F* < 1) or interaction, *F*(1, 9) = 1.11, *p* = 0.32, η^2^ = 0.11, with placeholder certainty on the estimated asymptote of the functions.

Similar to Experiment 1, we computed the AIC for selecting the model with the best fit (Table 2). We computed AIC for all the combinations of the threshold and asymptote parameters being constrained and unconstrained across the VSTM load and uncertainty conditions. Table 2 presents the different model parameterizations, and Table 3 presents the differences between the AIC values of the best-fitting model for each participant and the next three best-fitting models, as indicated by the average of the individual AIC values of each model. Model 1, which provided the best fit for eight out of 10 participants, was as suggested by the data fits in the analysis above—namely, it allowed the threshold and asymptote parameters to vary freely but assumed that the thresholds differed between low and high VSTM load conditions only in the uncertain condition and not in the certain conditions, whereas the asymptotes differed between the certain and uncertain conditions across VSTM load. Model 2, which provided the best fit for one out of 10 participants, assumed that, in the uncertain condition, high VSTM load would have an effect on both the threshold and the asymptote, in addition to a main effect of uncertainty on the asymptotes. Model 3, which did not provide the best fit for any of the participants, included no constrains on the thresholds and asymptotes, which were allowed to vary freely and to take on any value in any of the VSTM load and uncertainty conditions. Model 4, which provided the best fit for one out of 10 participants, was a fully restrictive model that assumed no differences between any of the conditions by allowing thresholds and asymptotes to vary freely but constraining them to be identical across all conditions. The AIC model selection criteria therefore provided support for the contrast gain account of VSTM load only in conditions in which there was demand on attention during memory maintenance (due to the location uncertainty of the orientation stimulus), as indicated by the average results.

**Table 2.** Contrast parameterization of the four models that provided the best fit according to AIC values (see Table 3 for ΔAIC values). *Notes*: U (unconstrained) indicates that a parameter estimate was free to vary and allowed to take on any value in any of the conditions. C (constrained) indicates that parameter estimates in all conditions were identical in value but this common value was a free parameter. Slopes and guess rates were fixed in all conditions of all models.

	Uncertain		Certain	
Model	Low load	High load	Low load	High load
Model 1				
Threshold	–0.33	1	–0.33	–0.33
Lapse rate	0.5	0.5	–0.5	–0.5
Model 2				
Threshold	–0.33	1	–0.33	–0.33
Lapse rate	−1	0.33	0.33	0.33
Model 3				
Threshold	U	U	U	U
Lapse rate	U	U	U	U
Model 4				
Threshold	C	C	C	C
Lapse rate	C	C	C	C

**Table 3.** ΔAIC values for the four models with the best fit for Experiment 2. *Notes*: For each participant, the difference (Δ) in AIC values is shown compared to the best-fitting model for that participant. Therefore, the ΔAIC value of the best-fitting model for each participant is zero (presented in bold).

	ΔAIC			
Participant	Model 1	Model 2	Model 3	Model 4
1	22.00	20.50	12.00	**0.00**
2	**0.00**	0.33	7.79	24.65
3	**0.00**	2.63	6.36	49.85
4	**0.00**	0.73	7.98	29.28
5	**0.00**	0.31	7.62	42.23
6	**0.00**	0.15	7.83	18.03
7	**0.00**	0.03	7.81	20.22
8	0.04	**0.00**	7.68	34.16
9	**0.00**	0.31	7.66	37.10
10	**0.00**	0.19	7.81	68.33

## Discussion

The present study investigated whether the effects of VSTM load on visual perception depend on shared attentional or sensory perceptual resources between the orientation discrimination task during the memory delay and the VSTM task. Increasing VSTM load affected orientation discrimination via a contrast gain mechanism as indicated by a VSTM load effect on the estimated contrast threshold of the contrast response function without affecting the asymptote of the function, as long as attention demands in the perception task were high. This finding indicates that VSTM load affects sensitivity to contrast in a manner that is akin to reduction of the apparent contrast of an irrelevant stimulus, suggesting a mechanism in which VSTM load leads to previous reports of impaired perception under conditions of high VSTM load (e.g., Konstantinou et al., 2012; Konstantinou & Lavie, 2013).

Importantly, these effects were found to depend on the level of demand on attentional resources required for contrast perception on the orientation discrimination task. The results of Experiment 2 establish that the effect of VSTM load on the contrast response function interacts with the specific demand on attention. High VSTM load exerted contrast gain effects only when the location uncertainty of the orientation stimulus was high, so that orientation detection required attentional resources for resolving the spatial uncertainty of the stimulus. These findings are informative about models of VSTM and specifically about the role of attention in VSTM. Below, we discuss each of these contributions.

### The effect of VSTM load on contrast perception

The present findings suggest that when resources involved in maintaining stimulus representations in VSTM are occupied in a high load task, the response to an unrelated stimulus of a given contrast is equivalent to the response to the same stimulus but of lower contrast. These effects are akin to the effects of perceptual load on the contrast response function (Lavie et al., 2014) and appear, on the face of it, to support the hypothesis that the source of perceptual failures with higher VSTM load is competition for sensory visual resources between VSTM and visual perception (Gayet et al., 2018; Harrison & Tong, 2009; Pasternak & Greenlee, 2005; Postle, 2006; Rademaker et al., 2019; Scimeca et al., 2018; Serences et al., 2009). This is because the level of contrast gain is known to be mediated by neural response in V1 (e.g., Ohzawa, Sclar, & Freeman, 1982); thus, the impact of VSTM load on the contrast gain suggests that VSTM load resulted in reduced V1 response to contrast. This interpretation is consistent with previous findings demonstrating the effects of VSTM load on V1 to V3 response to contrast. Konstantinou et al. (2012) demonstrated that VSTM load results not only in reduced detection sensitivity (*d*′) for a contrast increment target presented during the maintenance delay but also in reduced neural response to contrast increment in the retinotopic areas V1 to V3 corresponding to the stimulus presentation. As we discuss next, however, our findings that the effects of VSTM load on perception interact with the spatial uncertainty of the orientation discrimination stimulus point to a critical role of shared attentional resources in the effects of VSTM load on perception (and the related neural sensory response to contrast).

### Dependence of VSTM load effects on attentional resource demands

The interaction of VSTM load effect on the contrast gain and the cuing effect of spatial uncertainty manipulation that we established (Experiment 2) demonstrate that the effects of VSTM load on the contrast response function depend on the attentional demands placed on the orientation discrimination task during the VSTM maintenance. This is consistent with recent research that also reports sharing of attentional resources between VSTM and perception. For example, deBettencourt et al. (2019) found that performance in a sustained attention task requiring monitoring for a particular shape (e.g., square among circles) was correlated with performance on a concurrent VSTM task requiring maintenance of stimulus color, demonstrating that attention and VSTM resources co-fluctuate. Moreover, Balestrieri et al. (2019) replicated previous findings that higher VSTM load leads to reduced detection of an irrelevant stimulus during VSTM maintenance (Konstantinou et al., 2012; Konstantinou & Lavie, 2013) and that the presentation of the detection stimulus in various densely sampled time points during maintenance allowed assessment of the temporal oscillations in the effects of VSTM load on detection. Increasing VSTM load shifted the oscillatory detection pattern to lower fluctuation frequencies indicative of a trade-off in performance between visual detection and VSTM. Importantly, the findings showed that detection performance followed a temporal pattern similar to previous reports for temporal oscillations of attention (Busch & VanRullen, 2010; Landau & Fries, 2012; VanRullen, 2016; VanRullen, Carlson, & Cavanagh, 2007), in line with the hypothesis of shared attentional resources between perceptual representations and representations maintained in VSTM.

Our current results complement these previous findings in demonstrating the critical role of attention demand in the effects of VSTM load on perception of the orientation stimulus. Overall, the results are best accounted for as indicative of a competition for shared attentional resources between VSTM maintenance and sensory perception. The lack of evidence for a response gain effect also rules out an alternative account for the effects in terms of a mere change in top–down response bias, due to a general deprioritization of the detection task with increased load in the VSTM task. This conclusion is consistent with a previous finding that the effects of VSTM load on detection sensitivity during maintenance are only found when load is specifically increased in visual maintenance, while loading cognitive control resources in a verbal working memory task did not affect detection sensitivity (Konstantinou & Lavie, 2013).

### Temporal proximity account

Our proposed account that the effects of VSTM load on perception and the related neural response depend on the competition for attention resources can also explain an apparent discrepancy between the present findings and another body of work reporting the effects of temporal proximity on the interaction of VSTM and perception. Specifically, the present results demonstrate that VSTM load affects perceptual processing for a stimulus presented 1 second after the presentation of the memory set. This appears to be in contrast to a series of studies suggesting that interference effects on VSTM task performance that are produced by a visual distractor presented in the maintenance delay can only be found when the distractor is presented in temporal proximity to the memory set of less than 1 second. For example, Vogel, Woodman, and Luck (2006) found interference when the distractors were presented 117 ms after memory set offset but not when presented 584 ms after offset. van de Ven, Jacobs, and Sack (2012) used transcranial magnetic stimulation over the occipital cortex during memory delay and found negative effects on VSTM performance when stimulation was applied 200 ms after stimulus presentation but no effects at 400 ms. Recently, Xu (2017) interpreted these findings as being indicative of interference effects either during the consolidation process of visual information in VSTM or in preparation for the upcoming comparison of the memory probe to the information held in VSTM, suggesting that, when visual information has been consolidated in VSTM, perception and VSTM do not interact (for similar arguments, see also Bettencourt & Xu, 2015; Nemes, Whitaker, Heron, & McKeefry, 2011). However, although it is expected that a visual distractor stimulus can affect consolidation into VSTM, it is unclear how this account can explain the effects of VSTM load on contrast detection we report for a stimulus presented 1 second after the offset of the memory set, when consolidation process should have been complete (for similar or longer temporal separations, see also Konstantinou et al., 2012; Konstantinou & Lavie, 2013).

However, a difference in attentional demands between the different paradigms can explain the seeming contradiction between the findings that a distractor stimulus affects VSTM performance only when in temporal proximity to the memory set, whereas VSTM load affects visual perception even when the two sets of stimuli are temporally distinct. In the distractor interference paradigm, when observers are asked to ignore a visual stimulus presented in close proximity to the memory set, they are not required to perform a task on the distractor stimulus but rather to ignore it. It is plausible that a greater demand on attentional resources is required for filtering out distractors in temporal proximity to the memory set than filtering them when they are temporally distinct, as the temporal distinction can directly facilitate filtering.

On the other hand, here, despite the clear temporal distinction, attentional demand was induced by the visual orientation discrimination task during the delay period of a low or high load VSTM load task, as long as the location uncertainty was high. An interesting direction for future research would be to vary the level of attentional resources that the distractor attracts to examine whether this would interact with the effects of temporal segregation. It is possible, for example, that a more attention-captivating distractor will interfere even when temporally distinct from the VSTM set.

### Feature specificity account

We note that the effects we report here are neither feature specific nor spatially specific. Considering that the high VSTM load task that required maintenance of foveal color-location conjunction stimuli exerted effects of contrast gain for peripheral grayscale stimuli, these effects cannot be directly due to any form of shared feature-specific receptive field resources (c.f. biased competition) in primary visual cortex. Similarly, the impact on retinotopic response to peripheral contrast in V1 to V3 established for the same task as here (Konstantinou et al., 2012) cannot be attributed to any sharing of receptive field of V1 to V3 neurons. Further evidence from neuroimaging work indicates that attention can modulate the retinotopic V1 response in tasks that do not draw on the same feature-specific representations (e.g., Bahrami, Lavie, & Rees, 2007; Schwartz, Vuilleumier, Hutton, Maravita, Dolan, & Driver, 2005; Torralbo, Kelley, Rees, & Lavie, 2016). The fact that the effects do not require overlap in feature-specific representations further supports the suggestion that the impact of VSTM load on perception may be due to its impact on attentional resources rather than a direct impact on neural representations in V1.

Interestingly, recent findings suggest that sensory areas reflect memory representations of low-level basic visual attributes (e.g., luminance contrast), whereas more anterior areas in parietal and prefrontal cortex reflect more abstract or complex VSTM representations (Christophel, Klink, Spitzer, Roelfsema, & Haynes, 2017). Clarifying whether the effects of VSTM load on perception are confined to basic visual attributes (e.g., contrast, direction and speed of motion, spatial frequency) or can be extended to processing of more complex or abstract visual stimuli (e.g., faces) should be an interesting direction for future research.

### Relation to the normalization model of attention

The findings we report here are also consistent with the predictions of the normalization model of attention (Reynolds & Heeger, 2009). This computational model suggests that changing the size of the attention field in relation to the stimulus size determines whether directing attention to a stimulus will affect perceptual processing via contrast gain or response gain. When attention is directed to a stimulus and the relative size of the attention field is large, the model predicts contrast gain effects—that is, an effect of attention on the estimated contrast threshold of the contrast response function without affecting the asymptote. Here, we observed such contrast gain effects in Experiment 1 and in the uncertain condition of Experiment 2 when, indeed, the relative size of the attention field was large. Obtaining such effects predicted for attention but using VSTM load manipulation suggests that a similar mechanism is involved and provides further support for our proposal that the effects of VSTM load on perception are due to competition for attentional resources rather than sensory resources. The normalization model also predicts response gain effects when the relative size of the attention field is small. In our study, such response gain effects were observed (across the VSTM load conditions) in Experiment 2, where in the certain condition the spatial cue reduced the relative attention field size and resulted in response gain effects in line with the model predictions.

### Study limitations and future directions

The current study drew on the neurophysiological concepts of contrast gain and response gain to interpret the effects of VSTM load and attention on the contrast response function, assuming that such effects reflect different underlying neural mechanisms. However, our use of the Naka–Rushton model in its original formulation (e.g., Albrecht & Hamilton, 1982), which has been used in numerous studies, has the limitation that it does not allow us to directly relate our results to single neuron responses. A promising approach is offered by a model that includes components reflecting neural spikes and their variance (e.g., May & Solomon, 2015) in addition to the contrast response function. Future research examining the effects of attention and VSTM load on the contrast function using such a modeling approach should prove an important next step in our understanding of the impacts of attention and VSTM load on the neural processing of stimulus contrast.

In addition, our discussion of the effects of VSTM load and their dependence on attentional resource demands during perceptual processing also draws on the concept of limited-capacity neural resources being required for perception across different tasks. This leaves open the important question of the source of limited-capacity sensory neural resources and the role of attention in their allocation. Bruckmaier et al. (2020) recently offered a compelling neurophysiological account addressing this question in the case of perceptual load effects on neural response, attributing perceptual capacity limits directly to limits on cerebral cellular metabolism and proposing an attentional compensation mechanism that regulates cellular metabolism levels according to processing demands. Their work provides direct evidence for the effects of perceptual load on the cellular metabolism levels (as indicated by an intracellular measure of the metabolic enzyme cytochrome *c* oxidase) related to both attended and unattended processing. Future research applying such an approach to the effects of VSTM load on the neural response related to contrast perception can similarly substantiate the current conclusions that contrast perception critically depends on the level of overall attentional demand (both in the VSTM task and in the orientation perception task during the memory delay) on a limited neural resource that is required for stimulus perception.

## Conclusions

The present findings demonstrate that the effects of VSTM load on perception depend on the level of competition for attentional resources between VSTM maintenance and perception. When an orientation discrimination task during VSTM maintenance demanded attention, the effects of VSTM load on perception were consistent with a contrast gain mechanism. However, when demands on attention for the orientation discrimination task were reduced via a spatial cue, the contrast gain effects of VSTM load were eliminated. These findings clarify the effects of VSTM load on visual perception for a temporally distinct stimulus during maintenance and support accounts of attentional resource sharing between VSTM maintenance and perception.
